# Unveiling the Veiled: Enteroviral Meningitis Mimicking Idiopathic Intracranial Hypertension

**DOI:** 10.7759/cureus.63884

**Published:** 2024-07-05

**Authors:** Sathvika A, Nirmala D C, Shiny PJ, Sreevinishaa Ravichandran, J S Kumar

**Affiliations:** 1 General Medicine, Sri Ramaswamy Memorial (SRM) Medical College Hospital and Research Centre, Sri Ramaswamy Memorial (SRM) Institute of Science and Technology, Chengalpattu, IND; 2 Medical Research, Sri Ramaswamy Memorial (SRM) Medical College Hospital and Research Centre, Sri Ramaswamy Memorial (SRM) Institute of Science and Technology, Chengalpattu, IND

**Keywords:** atypical presentation, central nervous system infections (cns), intracranial pressure, pseudotumor, enteroviral meningitis

## Abstract

Meningitis is a significant health concern globally, with enterovirus (EV) being the most common cause of viral meningitis in adults. We discuss the case of a 57-year-old female patient with enteroviral meningitis manifesting as pseudotumor cerebri, posing significant clinical challenges. She presented with symptoms, signs, and radiological evidence suggesting idiopathic intracranial hypertension. The CSF analysis showed pleocytosis, which led to further investigations that unveiled a positive case of EV by real-time reverse transcription polymerase chain reaction analysis. This case highlights the fact that not all cases of raised intracranial pressure are detrimental or recalcitrant. It accentuates the need for thorough diagnostic evaluation and emphasizes the potential for favorable outcomes with conservative management.

## Introduction

Pseudotumor cerebri, also known as idiopathic intracranial hypertension (IIH), is characterized by elevated CSF pressure (>25 cm H2O), normal CSF laboratory findings, and unremarkable radiographic scans. Individuals with this condition often experience a variety of symptoms, including headaches, nausea, papilledema, diplopia, and visual field deficits [1].

Enteroviral meningitis is caused by various serotypes of enterovirus (EV), with echovirus and coxsackievirus (CV) being the most common CNS infections due to meningeal inflammation. Enteroviral meningitis is typically transmitted through fecal-oral or respiratory routes [2]. Patients typically present with symptoms of fever, headache, photophobia, neck stiffness, and altered consciousness.

Here, we present a case in which the patient had enteroviral meningitis confirmed by real-time reverse transcription polymerase chain reaction (RT-PCR), displaying features of increased intracranial pressure (ICP) that posed a diagnostic challenge. This case highlights the importance of considering viral causes in patients presenting with IIH symptoms, especially when accompanied by CSF pleocytosis.

## Case presentation

A 57-year-old female homemaker presented to the general medicine OPD with complaints of dizziness (spinning sensation). Each episode lasted for three to five minutes, with multiple episodes occurring throughout the day over the past two weeks. These episodes were intermittently accompanied by bilateral tinnitus. She also reported a dull, diffuse headache persisting for two weeks, occasionally associated with vomiting one to two times per day containing food particles. She had visited the ENT OPD a week prior for similar complaints and was initially diagnosed with benign paroxysmal positional vertigo (BPPV). Treatment with betahistine and nonsteroidal anti-inflammatory drugs was initiated, but due to worsening symptoms, she sought further evaluation in the medicine OPD. The patient denied any history of fever, neck stiffness, memory loss, or loss of consciousness. She had a medical history of type 2 diabetes mellitus and systemic hypertension for the past five years, which she managed with regular medication and compliance. She did not use herbal medication or vitamin supplements, and she was not a smoker or alcoholic. She had no history of recent travel, and none of her family members were experiencing similar complaints.

On examination, the patient was conscious and oriented to time, place, and person; her pulse was regular at 86 beats per minute; her blood pressure was 120/70 mmHg; her temperature was 98.8F; and her oxygen saturation was 97% on room air. Her blood glucose is 145 mg/dl, and her Glasgow Coma Scale is 15/15. Fundus examination showed blurring of the nasal disc margin, suggesting early bilateral papilledema. There were no signs of cerebellar involvement or orthostatic hypotension noted. Examination of other systems was normal. Considering the possibility of a posterior circulatory stroke due to swaying observed during gait examination, an MRI of the brain was performed. The results of the biochemical parameters from blood and CSF are presented in Table 1.

**Table 1:** Results of the biochemical parameters from blood and CSF The high opening CSF pressure and CSF leukocytosis indicate elevated ICP and underlying pathology. ICP, intracranial pressure; LDH, lactate dehydrogenase

Parameters	Result	Reference
Blood		
Hemoglobin	11.8 g/dL	12-16 g/dL
WBC count (cells/mm^3^)	12,440	4,000-11,000
Neutrophil	63%	40-70%
Lymphocyte	22%	20-40%
Platelets	327,000 cells/mm^3^	150,000-400,000 cells/mm^3^
CSF		
Opening pressure	33 cm H_2_0	8-18 cm H_2_0
Appearance	Clear	Clear
WBC count	105 cells/mm^3^	0-5 cells/mm^3^
WBC (lymphocytes)	85%	Predominantly lymphocytes
WBC (neutrophils)	15%	0%
RBC count	13 cells/mm^3^	0 cells/mm^3^
Protein	105.7 mg/dL	15-45 mg/dL
Glucose	35 mg/dL	50-75 mg/dL
LDH	35 U/L	Less than 40 U/L
Chloride	125 mg/dL	118-132 mg/dL

Antinuclear antibodies and Mantoux were found to be negative. The CSF culture sensitivity result showed no growth; the cartridge nucleic acid amplification test for tubercular bacilli was negative; and the acid-fast bacilli (AFB) stain was negative. In view of elevated lymphocytes in the CSF, a meningoencephalitis panel was tested by real-time RT-PCR, which was reported to be positive for viral EV, while cytomegalovirus, herpes simplex virus (HSV) 1,2, human herpes virus, human parechovirus, and varicella zoster virus were seen to be negative. Bacterial and fungal tests for *Haemophilus influenzae*, *Streptococcus agalactiae*, *Streptococcus pneumoniae*, and *Cryptococcus neoformans* or *Cryptococcus gattii *were reported as negative. RT-PCR results for Mycobacterium tuberculosis and non-tuberculous mycobacteria were also negative.

**Figure 1:**
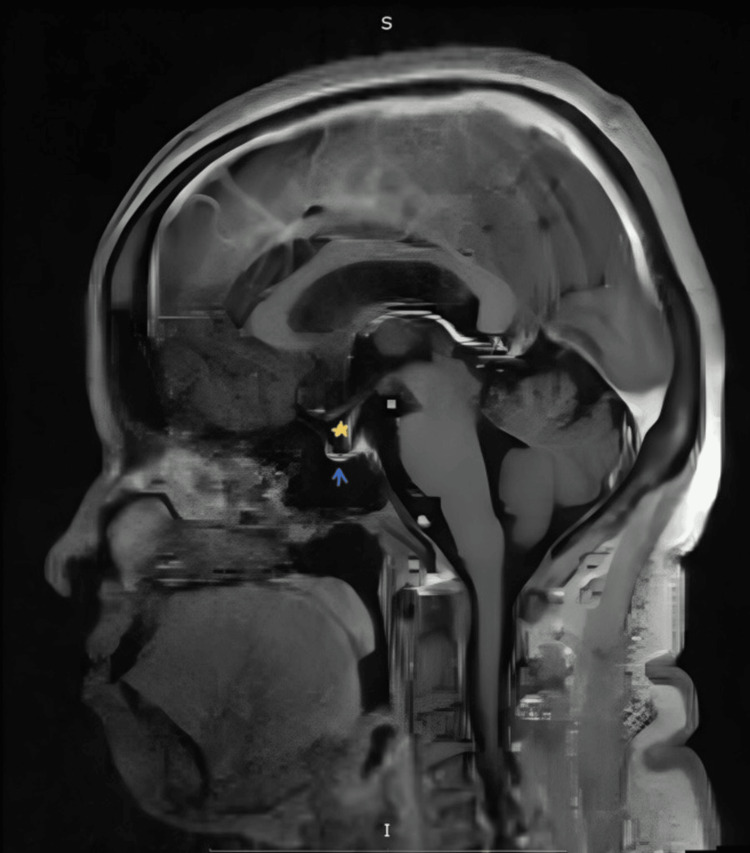
Sagittal section contrast MRI of the brain illustrating the flattening of the pituitary gland (blue arrow) and a partially empty sella (yellow star)

**Figure 2:**
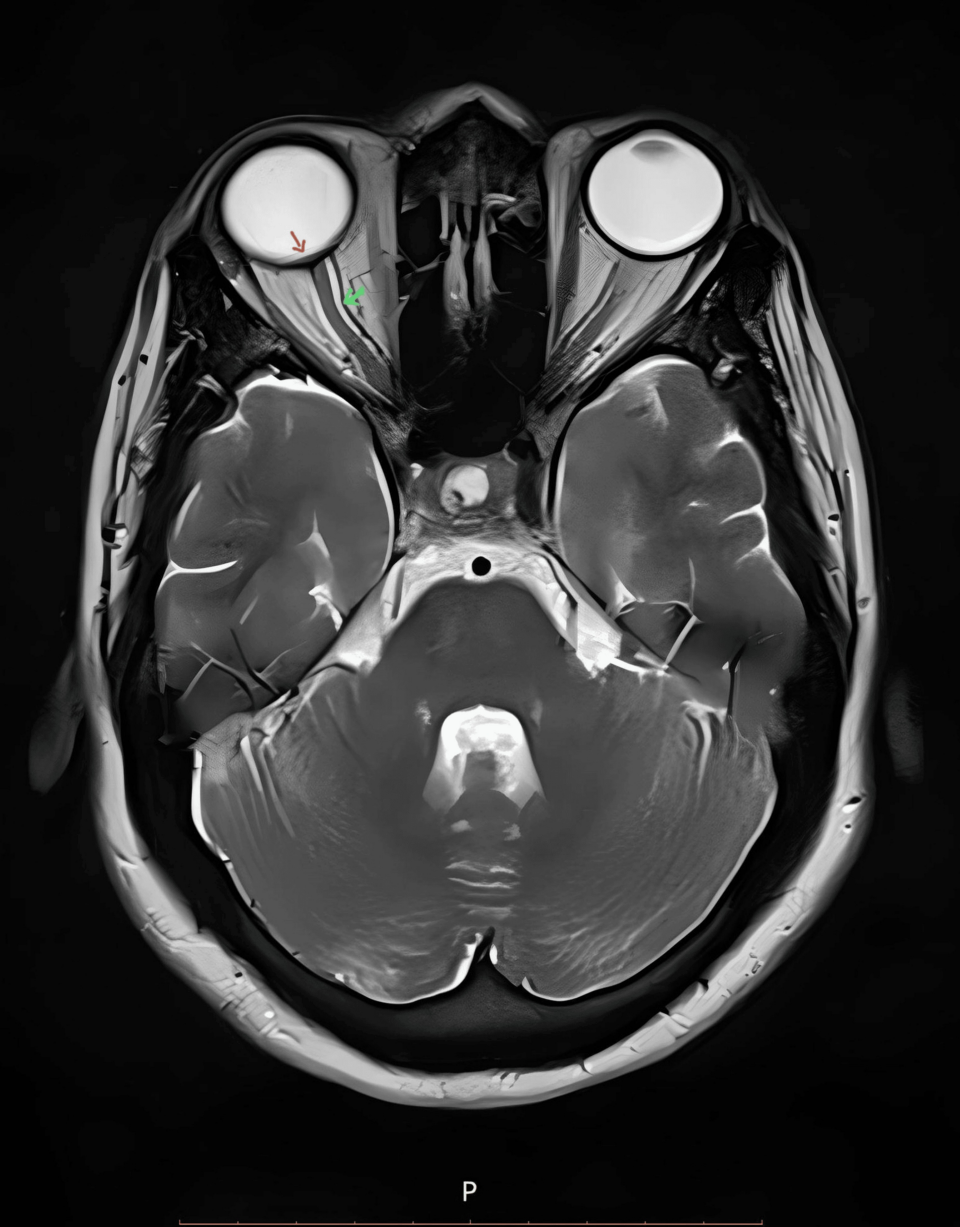
Axial T2-weighted MRI of the brain at the level of the optic nerve, displaying hyperintense CSF around the optic nerve (green arrow) and bulging of the optic nerve papillae (red arrow), indicative of papilledema

The radiological investigations revealed a normal chest X-ray. The MRI brain with angiography and venography with whole spine screening was done, which revealed features suggesting IIH such as an empty sella with perioptic nerve sheath CSF space prominence and mild tortuous orbital segments of bilateral optic nerves (Figures 1, 2).

Throughout her hospital stay, differential diagnoses considered included BPPV, posterior circulatory stroke, IIH, and other potential causes such as viral meningitis, HSV, human herpesvirus, HIV, fungal meningitis (histoplasmosis), cryptococcal, tuberculous meningitis, autoimmune meningitis, and arteriovenous malformations.

Based on clinical and radiological findings consistent with pseudotumor cerebri, the patient received treatment with inj mannitol 100 mg once daily and T-acetazolamide 250 mg twice daily for two days. After the CSF report showed lymphocyte-predominant leukocytosis suggestive of viral or tuberculous meningitis, prophylactic treatment was initiated with inj acyclovir 500 mg every eight hours and inj dexamethasone 8 mg IV every 12 hours for two days. Tab indomethacin (NSAID) was administered for analgesia. Steroids were discontinued after the viral panel returned positive for enteroviral meningitis, and antivirals were continued for five days. Symptoms resolved within seven days of treatment. The patient’s condition remained stable at discharge, and she is currently on follow-up without experiencing any new symptoms.

## Discussion

EV infections are a significant global health concern, causing substantial illness and death. Belonging to the picornavirus family, the EV genus includes well-known viruses like poliovirus, CV, and EV-71. Non-polio EVs are capable of affecting the CNS, leading to various clinical conditions such as encephalitis and meningitis. The long-term effects of EV infections on the CNS remain poorly understood, although these viruses can persist, and the presence of viral RNA alone has been implicated in potential pathogenicity in certain cases [2]. Molecular methods such as RT-PCR are highly sensitive and precise in detecting EV infections in the CNS, with CSF being the preferred clinical sample for analysis.

In this case, gene expression analysis using RT-PCR was done within 24 hours of sample collection. Bacterial and fungal meningitis are well known for causing raised ICP due to cytotoxic and interstitial edema, resulting from increased permeability of the blood-brain barrier [3]. They also induce inflammation and edema within the meninges, leading to obstruction of CSF flow and impaired absorption. Consequently, the accumulation of CSF within the ventricular system and subarachnoid space contributes to increased pressure within the intracranial cavity. While increased ICP due to viral meningitis is extensively documented in the literature, there have been case reports of viral meningitis presenting as IIH [4].

This patient presented with features suggesting pseudotumor cerebri and meets the modified Dandy-Walker criteria. Signs and symptoms include headaches, often worse in the morning, pulsatile tinnitus, nausea, vomiting, papilledema, and visual disturbances. Elevated ICP with an opening pressure of >25 cm H_2_O is noted in the absence of an intracranial mass lesion. CSF analysis shows normal cell count, glucose, and protein levels, ruling out infectious or inflammatory etiologies. Neuroimaging studies, including MRI and CT scans, should reveal no structural abnormalities causing increased ICP. The ventricles may appear normal in size or slightly compressed due to increased pressure [5-7]. Viral meningitis associated with increased ICP, fever, and other signs of meningitis has been reported in very few cases. However, to our knowledge, the isolated presentation of increased ICP with EV is unique [8].

During the assessment, the absence of infection indicators such as fever, elevated white blood cell counts, normal erythrocyte sedimentation rate, CRP, and leptomeningeal enhancement in MRI was akin to chasing elusive shadows in the realm of diagnostic uncertainty. Nevertheless, recent case reports suggest a potential link between viral meningitis and intracranial hypertension, hinting at the possibility of identifying other causes of intracranial hypertension through improved diagnostic methods [9-11]. EV-induced meningitis typically resolves on its own in immunocompetent individuals without specific treatment, although the use of antivirals in these cases remains uncertain. Initiating antiviral therapy should be guided by clinical assessment to prevent potential neurological complications.

## Conclusions

While viral meningitis is common, encountering such a presentation in an immunocompetent patient is rare. In the context of IIH, even without overt symptoms of meningitis and the absence of leptomeningeal enhancement on MRI, it is advisable to consider sending a viral panel. This approach can assist in ruling out viral meningitis, thereby avoiding unnecessary hospitalization and additional investigations.

## References

[1] Kiefer L, Adam D, Mudugal D, Burnett MS (2020). Viral meningitis mimicking benign intracranial hypertension: a report of two cases. Interdiscip Neurosurg.

[2] Rhoades RE, Tabor-Godwin JM, Tsueng G, Feuer R (2011). Enterovirus infections of the central nervous system. Virology.

[3] Kim D, Small JE (2019). Idiopathic intracranial hypertension (pseudotumor cerebri). Neuroradiology.

[4] Quagliarello V, Scheld WM (1992). Bacterial meningitis: pathogenesis, pathophysiology, and progress. N Engl J Med.

[5] Dandy WE (1937). Intracranial pressure without brain tumor: diagnosis and treatment. Ann Surg.

[6] Friedman DI, Jacobson DM (2002). Diagnostic criteria for idiopathic intracranial hypertension. Neurology.

[7] Wall M (2008). Idiopathic intracranial hypertension (pseudotumor cerebri). Curr Neurol Neurosci Rep.

[8] Beal JC (2017). Increased intracranial pressure in the setting of enterovirus and other viral meningitides. Neurol Res Int.

[9] Ravid S, Shachor-Meyouhas Y, Shahar E, Kra-Oz Z, Kassis I (2012). Reactivation of varicella presenting as pseudotumor cerebri: three cases and a review of the literature. Pediatr Neurol.

[10] Sherchan R, Shrestha J, Omotosho YB, Dyatlova N, Nepomuceno JS (2021). Herpes simplex virus-2 meningitis masquerading as pseudotumor cerebri. Cureus.

[11] Smith HZ, Paguia R, Horne J, Velagapudi M (2019). A case report of human herpesvirus-6 (HHV-6) meningitis masquerading as idiopathic intracranial hypertension in an immunocompetent patient. Cureus.

